# The district operation centres in one of the largest local health authorities in Italy to manage COVID-19 surveillance and homecare: first implementation and results of a survey addressed to general practitioners

**DOI:** 10.1186/s12913-023-10213-3

**Published:** 2023-11-07

**Authors:** Emanuela Maria Frisicale, Andrea Barbara, Alessio Perilli, Elettra Carini, Adriano Grossi, Leonardo Simonetti, Giulia Tammam, Svetlana Axelrod, Angelo Tanese, Mauro Goletti, Paolo Parente

**Affiliations:** 1 Directorate General of Health Prevention, Ministry of Health, Rome, Italy; 2 Department of Public Health and Infectious Diseases, Sapienza University of Rome, Rome, Italy; 3 Local Health Authority Roma 1, Rome, Italy; 4 Department of Life Sciences and Public Health, Hygiene Section, Università Cattolica del Sacro Cuore, Rome, Italy; 5 World Health Organization, Geneve, Switzerland; 6 First Sechenov University, Moscow, Russia

**Keywords:** COVID-19, Primary healthcare, Organizational innovation, Change management, Process evaluation

## Abstract

**Background:**

COVID-19 pandemic represented a shock for healthcare systems. Italy was one of the first country to deal with a huge number of patients to be diagnosed, isolated, and treated with scarce evidence-based guidelines and resources. Several organizational and structural changes were needed to face the pandemic at local level. The article aims at studying the perceived impact of the newly implemented District Operation Centres (DOCs) of Local Health Authority (LHA) Roma 1 in managing active surveillance and home care of COVID-19 patients and their close contacts in cooperation with general practitioners (GPs).

**Methods:**

A questionnaire, developed according to Delphi methodology, was validated by 7 experts and administered to a randomized sample of GPs and family paediatricians (FPs). All medical doctors selected received a phone interview between December 2020 and January 2021. The questionnaire investigated general characteristics of the sample, relations with DOC and its usefulness, and potential developments. A descriptive analysis was performed and inferential statistical tests were used to assess differences.

**Results:**

In April 2020 the LHA Roma 1 implemented one DOCs in each local health district. 215 medical doctors were interviewed, reaching the sample target for health districts (80% CL and 10% MOE) and the whole LHA (90% CL and 5% MOE). Several aspects in the management of COVID-19 cases and close contacts of COVID-19 cases, and of the support of DOCs to GPs/FPs were investigated. More than 55% of the GPs and FPs interviewed found the DOCs useful and more than 78% would recommend a service DOC-like to other LHAs. The medical professionals interviewed would use DOCs in the future as support in treating vulnerable patients, utilizing digital health tools, enlisting specialist doctors, establishing networks, and facilitating professional counselling by nurses.

**Conclusions:**

This study is an attempt to evaluate an organizational change happened during COVID-19 pandemic. DOCs were created to support GPs and FPs as a link between primary healthcare and public health. Although several difficulties were disclosed, DOCs’ experience can help to overcome the fragmentation of the systems and the duality between primary care and public health and make the system more resilient.

**Supplementary Information:**

The online version contains supplementary material available at 10.1186/s12913-023-10213-3.

## Introduction

COVID-19 is an infectious disease caused by SARS-CoV-2, which was first detected in Wuhan, China in December 2019 [1]. It rapidly spread across the world, being defined as a pandemic by WHO in March 2020 [2]. Nowadays, millions of cases and deaths related to COVID-19 have been reported worldwide [3].

Governments deployed a series of measures to various degrees of stringency to contain the outbreak [4–6]. They included social distancing, smart working, mass masking, curfew, business closures, and travel bans, which proved to be effective [7–9].

Health systems were directly exposed to a massive shock, as they struggled to deal with an outstanding number of patients to diagnose and treat, in a scarcity of evidence-based treatments [10] and available resources [11, 12]. Health workforce shortages, broken supply chains, fragmented services and silo information systems are among the underlying issues that were brought to light by the COVID-19 emergency [13, 14]. Nonetheless, health systems often rose to the task, proving they were able to rapidly change and innovate [15], reinforcing the overall health systems’ resilience capability [16].

The Italian health system was particularly affected by COVID-19 epidemic [17], with a great impact on both primary and secondary care and, in general, on the organisational aspect of Local Health Authorities (LHAs) and hospitals in the whole country [17–19].

Within the Italian National Health Service (INHS), LHAs are public entities in charge to organise and deliver healthcare services to the population through their own facilities or in collaboration with private providers. LHAs provide primary care/family medicine, public health and secondary care services through their different facilities (also hospitals) dislocated in their territory. Each LHA is divided in local health districts (LHDs) which have the governance of primary care services provisions at the local level working together with the municipal authorities in order to guarantee integrated social care services. Public health services and preventive medicine are delivered by the LHA Public Health Department. Mental health services are provided by the specific Department too [20].

During a pandemic infection, primary care represents the first line of defence and plays a relevant role in the response: identifying potential cases, making a diagnosis, reinforcing patients’ and citizens’ compliance to prevention and public health measures, supporting the management of patients at home, identifying who needs hospital care [18, 19, 21, 22]. Thus, primary care enhancement, specifically, was needed to provide homecare to COVID-19 cases, alleviating pressure on hospitals, as already showed by Serafini et al. [23]. For this reason, among the innovations introduced, the District Operational Centres (DOCs) were implemented at a local level [24]. These centres were designed to manage the active surveillance and homecare of COVID-19 patients - suspected and confirmed cases and close contacts of COVID-19 cases - in cooperation with General Practitioners (GPs) and Family Paediatricians (FPs) [24]. Moreover, to provide home care for people who tested positive for SARS-CoV-2, “special continuity of care units” (Unità Speciali di Continuità Assistenziale - USCA) were created according to national and regional laws and were composed of professionals (doctors and nurses) that provided at-home non-urgent care [25, 26]. At the same time, digital health implementation took place to face the emergency [27, 28]. Conversely, ambulatory services were suspended [29].

This study aims to describe shortly the first implementation of the DOCs in the LHA Roma 1 and assess their perceived utility and the degree to which GPs and FPs appreciated the new establishment, in an attempt to understand how impactful they are from a resilience perspective. In order to pursue this objective a survey was created and administered by phone to the GPs and FPs of the LHA after almost 1 year of DOCs implementation.

## Materials and methods

### Setting

The LHA Roma 1 is one of the biggest LHAs in Italy [30], delivering healthcare services to more than 1.000.000 inhabitants in a 524 km^2^ area within the city of Rome [31]. LHA Roma 1 comprises clinical departments -in hospital and ambulatory services-, six local health districts -LHD 1, 2, 3, 13, 14, and 15, according to the municipality of Rome, which mainly manage primary health care-, a mental health department and a prevention department, in which the public health unit is embedded [31].

DOCs were implemented in the LHA Roma 1, one in each LHD and were designed to manage the active surveillance and home-care of COVID-19 patients - suspected and confirmed cases and close contacts of COVID-19 cases - in cooperation with GPs and FPs [24].

### Study design

In order to assess the DOCs’ effectiveness and the degree to which GPs and FPs appreciated the new establishment, we conducted a cross-sectional study among the GPs/FPs of the LHA Roma 1, developing and administering them a survey by phone.

### Survey development and Delphi assessment

A questionnaire was developed by a team made up of researchers, medical doctors of DOCs and the prevention department and the medical directors of the LHA.

The Delphi methodology was followed to validate the questionnaire [32, 33]. As a minimum number of experts is not defined [32, 33], the questionnaire was sent to 7 experts: 2 public health physicians with a management background, 2 directors of two LHDs of the Roma 1 LHA, 2 GPs of the Roma 1 LHA who contributed to devising the DOCs and 1 GP affiliated with a different LHA. The directors of the two LHDs and the two GPs working in the Roma 1 LHA were chosen because they joined the LHA COVID-19 primary health care response task force, in charge to organise and plan primary care services delivery to face this extraordinary situation [34]. The two public health experts were chosen because of their experience in research and in organisation and evaluation of primary healthcare services.

Two rounds of consultations were carried out in order to provide the final survey, composed by 21 questions mainly concerning GPs/FPs’ interaction with DOCs, their assessment of DOCs’ usefulness both during, before and after the pandemic, and their use of other health district services. Questions’ answers were based on a five-point Likert scale, one of the most used [35].

More details about the process of drafting the questionnaire are provided in the supplementary material (Supplement 1), which includes the final version of the questionnaire.

### Sample size determination, sample selection and randomization

As of October 30, 2020, Roma 1 LHA included 854 GPs and 130 FPs, unevenly distributed among the six health districts (Table S2.1).

Sample size was determined per health district using Epi Info® considering effective a number of responses with an 80% (or higher) Confidence Level (CL) and a 10% (or lower) Margin of Error (MOE), in order to not lose the strength of the information obtained on a district level and taking into account the sustainability of the survey dissemination. More details of the sample size determination are reported in supplementary material (Supplement 2).

Following sample size determination, sample randomization without repetition by means of EXCEL (Microsoft Corporation, US) RAND function was performed for each district to causally select which GP/FP had to be interviewed first to reach the requested sample size. A maximum of three attempts of contact was done for each selected physician before moving on to another physician further down the randomized list, until the minimum target sample size was achieved.

### Survey dissemination and GPs and FPs enrollment

A mail describing the initiative was sent by the medical directorate of Roma 1 LHA to all GPs and FPs in order to increase the adherence to fill the survey through a phone interview. Interviews were performed simultaneously in the six LHDs and were entirely carried out by telephone by three medical doctors. The participation of GPs and FPs was voluntary and unpaid. Informed oral consent was requested from all the participants, and it was necessary to participate in the survey.

### Statistical analysis

The analysis of the responses was mainly descriptive. For each response at a district level, we presented continuous variables, including the five-point Likert scale [36], as median and 25° (Q1) and 75° (Q3) percentile, interquartile range (IQR), or mean and standard deviation, while categorical variables as number and proportion. When present, missing data were excluded from the analysis. To compare differences, we used inferential statistic test, as independent group t-tests or the Mann‐Whitney U test, when appropriate, and χ² test or the Fisher’s exact. The software used was STATA ver 15.0 (StataCorp LLC).

Logistic regression and collinearity statistic were performed using Jamovi software (version 1.6) to analyse the relationship between age (in years), sex, the number of managed patients per capita, job description (GP or FP), years of practice (more than 20 years, between 5 and 20 years, less than 5 years; the cut-off was arbitrarily chosen but with the willing to consider the seniority level), working districts (LHD 1, 2, 3, 13, 14, 15) and the GPs’ and FPs’ perception of how useful DOCs were. This last point refers to the survey´s question “Overall, how do you rate the DOC usefulness?”, where only answers higher or equal to 4 on the 5-points Likert scale were labelled as “useful”. P-value < 0.05 was considered statistically significant.

### Geospatial reference

Data concerning the GPs/FPs’ main medical offices addresses were processed through QGIS 3.16.2-Hannover (MMQGIS plugin), to compose a map displaying the spatial distribution of the interviewed GPs/FPs’ main medical offices. Results are in supplementary materials (Supplement 2).

## Results

### DOCs first implementation and organizational model

**Fig. 1:**
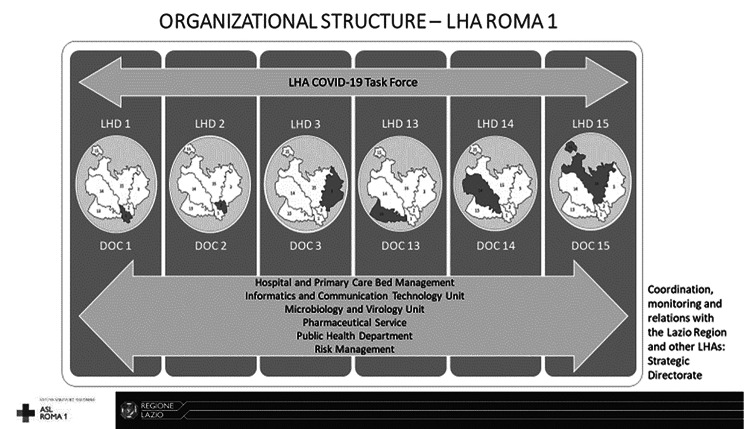
The organizational structure of the LHA Roma 1 to manage the active surveillance and home-care of COVID-19 patients - suspected and confirmed cases and close contacts of COVID-19 cases – with the main services involved and the crucial role of the newly implemented DOCs.

In April 2020 the LHA Roma 1 implemented one DOC in each health district and, according to the population served by the district, a minimum number of professionals - medical doctors, nurses, social workers and administrative staff - was distributed [24]. Each LHD designated a contact person working in the DOC as responsible for the DOC development and its workload, always connected with the other members of the emergency task force and GPs and FPs (Fig. 1) [34]. In addition, an on-the-job training course was held for all the LHA contact persons, GPs and FPs to illustrate the functions, organization and management methods of the newly implemented services [24].

At the end of 2020, more than 30,000 COVID-19 cases were registered in the LHA Roma 1 [37]. The majority of these and their close contacts were managed by the DOCs in collaboration with GPs and FPs.

### Survey results

#### Comparison between interviewed and non-interviewed subjects

Interviews started in the last decade of December 2020 and finished in the first half of January 2021 when the targeted sample size was reached. Overall, 215 subjects (corresponding to 21.9% of all GPs/FPs of LHA) were interviewed.

No differences were found between population sampled/interviewed and the remaining population not interviewed for some main characteristics, except for the gender variable (the female gender was more represented in the sample) (Table 1).

**Table 1:** Description of the interviewed sample, as compared to the remaining population

Variable		Interviewed	Not interviewed	p value
**Total number** – n (%)		215 (21.9%)	769 (78.1%)	
**District distribution** (number of physicians per District) – n (%)	District 1 (195)	36 (18.5%)	159 (81.5%)	0.15
	District 2 (190)	39 (20.5%)	151 (79.5.0%)	
	District 3 (194)	36 (18.6%)	158 (81.4%)	
	District 13 (125)	36 (28.8%)	89 (71.2%)	
	District 14 (162)	36 (22.2%)	126 (77.8%)	
	District 15 (118)	32 (27.1%)	86 (72.9%)	
**Job**	GPs	179 (21%)	675 (79%)	0.08
	FPs	36 (27.7%)	94 (72.3%)	
**Number of managed patients by each physician**				
Overall Median (Q1-Q3)		1028 (732–1507)^a^	1092 (685–1496)^b^	0.87
GPs Median (Q1-Q3)		1139 (757–1522)	1152 (680–1511)	0.68
FPs Median (Q1-Q3)		800 (715–849)	788 (705–850)	0.47
**Physician age in years**				
Median (Q1-Q3) (mean ± DS)		61 (57–65) (59.1 ± 8)	62 (57–66) (59.7 ± 8.6)	0.04
**Gender**	Female	122 (25.2%)	362 (74.8%)	0.01
	Male	93 (18.6%)	407 (81.4%)	

^a^ 4 missing; ^b^ 32 missing

### Description of main results

153 (71.2%) GPs and FPs interviewed have been working as such for more than five years.

Describing what the interviewed GPs and FPs knew about the DOCs, 182 (84.7%) GP/FPs declared to know the DOCs since their creation in April 2020 and this information was spread more in district 13 than in the other ones (although the difference is not statistically significant). The most used channel of communication by GPs and FPs to contact the DOCs was email and this contact resulted to be constant.

Concerning the orientation to the regulation and to the certificates to release by GPs/FPs (e.g. certificate of recovery), DOCs had a discrete role (mean on a 5-point Likert scale: 3.41) in supporting healthcare workers navigating these themes. In particular, about the orientation to the regulation a significant difference was found for DOC 2 when compared to the other DOCs: the healthcare workers of district 2 seem to have been less supported in the orientation to regulation than the other districts (p < 0.05).

Regarding the management of people diagnosed positive to the SARS-CoV-2, several sub-dimensions were investigated to better describe the process of management from the identification of the infection until the release of certificates or the management of cohabitants. Each sub-dimension under investigation had a mean that was less than 3 on a 5-point Likert scale, except for one dimension (“activation of USCA to perform COVID-19 tests”) for which the mean was 3.11. Differences among the six districts were minimal, even if in some cases statistically significant. The same results emerged when the management of clusters was investigated (mean on a 5-point Likert scale: 2.35), where DOC 15 seems to have performed worse than the other DOCs (mean: 1.69) while DOC 13 performed better (mean: 2.92). Moreover, for the management of close contacts, it emerged that DOCs were not of support.

Investigation of the dimensions “support in the relationship with the public health unit” and “support for the use of regional digital tools” revealed low scores (mean on a 5-point Likert scale: 2.65 and 1.83, respectively). On the other hand, the support to activate the teams in charge for executing swabs was relevant (mean on a 5-point Likert scale: 3.11), while the support in organizing home medical consulting by the same teams was not relevant (mean: 2.35). The support of the districts in providing home care interventions or consulting was even scarce (mean: 1.54) with no differences among the six districts.

**Fig. 2:**
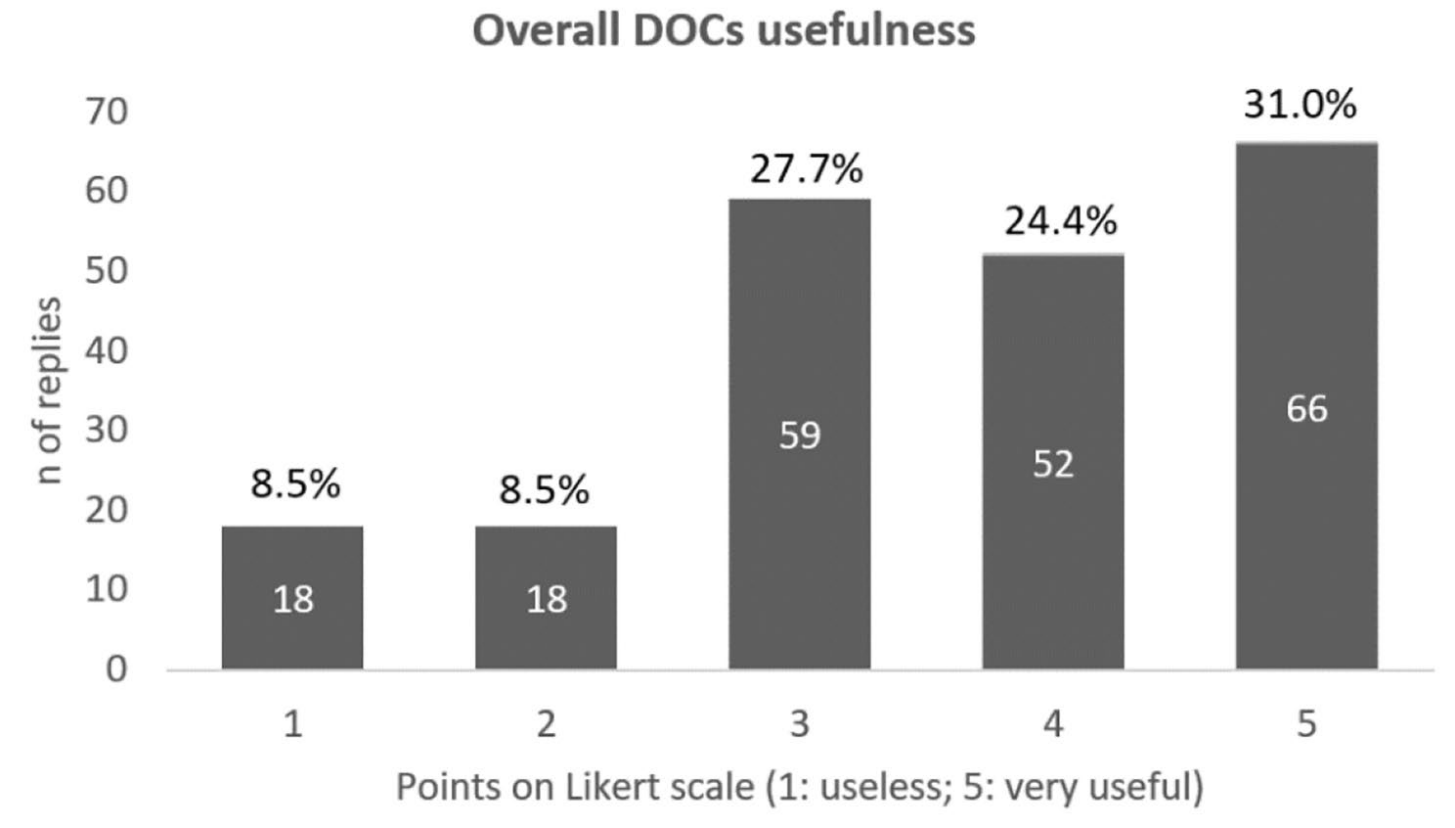
Frequency of replies to question “Overall, how do you rate the DOC usefulness?” divided by Likert scale score (from 1 to 5). Number of replies = 213

Overall, more than 55% of GPs and FPs interviewed considered useful the creation of the DOCs (118 out of 213 scored 4 to 5 on a 5-point Liker scale, Fig. 2) and more than 78% would suggest a service DOC-like to other LHAs (168 out of 214 scored 4 to 5), with minor improvements (e.g. dedicated telephone number/email, further availability or provision of home care are needed).

Performing the logistic regression model to ascertain the effects of age, sex, the number of managed patients, years of practice, job description and working districts on the likelihood that physicians consider the DOC useful, no variable with a value of VIF > 10 was detected; the highest value was 1.48 for age (years). The model explained 9.3% (Nagelkerke R2; Cox & Snell’s R2 was 6.9%) of the variance in considering useful the DOC and correctly classified 64.6% of cases. As shown in Table 2, working in District 3 (vs. District 13) significantly reduced the probability of considering useful the DOC by the physician.

**Table 2:** Logistic regression predicting the likelihood of considering useful the DOC by the physician

	Estimate	95% Confidence Interval		Odds Ratio	95% Confidence Interval		SE	Z	*P*
		Lower	Upper		Lower	Upper			
**Intercept**	0.53	-2.11	3.16	1.69	0.12	23.64	1.35	0.39	0.70
**Age (years)**	-0.01	-0.06	0.04	0.99	0.94	1.05	0.03	-0.27	0.79
**Sex**									
F	1								
M	-0.11	-0.70	0.49	0.90	0.50	1.63	0.30	-0.35	0.73
**Number of managed patients**	4.07e-4	-3.23e − 4	0.00114	1.0	1.0	1.0	3.73e-4	1.09	0.28
**Years of practice**									
< 5 y	1								
5–20 y	0.45	-0.66	1.56	1.57	0.52	4.76	0.57	0.79	0.43
> 20 y	0.01	-1.25	1.26	1.01	0.29	3.51	0.64	0.01	0.99
**Job type**									
GP	1								
FP	0.88	-0.07	1.83	2.42	0.94	6.24	0.48	1.82	0.07
**District**									
13	1								
1	-0.38	-1.40	0.64	0.69	0.25	1.90	0.52	-0.73	0.47
2	-0.91	-1.89	0.07	0.40	0.15	1.08	0.50	-1.81	0.07
3	-1.07	-2.08	-0.07	0.34	0.13	0.94	0.51	-2.09	0.04
14	-0.26	-1.29	0.75	0.77	0.28	2.13	0.52	-0.51	0.61
15	-0.38	-1.44	0.68	0.69	0.24	1.98	0.54	-0.70	0.48

Note. Estimates represent the log odds of usefulness of the CODs by the physician (useful = 1 if replies to question “Overall, how do you rate the DOC usefulness?” was higher or equal to 4; not-useful = 0 if 3 or less)

Regarding the future, the medical doctors interviewed were interested in implementing DOCs to be supported in managing frail patients (mean on a 5-point Likert scale: 4.44) and using digital health tools (mean on a 5-point Likert scale: 3.75). At the same time, they retain that DOCs could be useful for involving specialist doctors and building networks with GPs (mean on a 5-point Likert scale: 4.3) and for delivering counselling by professionals as nurses (mean on a 5-point Likert scale: 4.1).

High scores in specific questions of the survey revealed that GPs and FPs would like to be involved in some districts’ activities such as planning and improving clinical pathways or those regarding prevention and health promotion. All related data and all the results are shown in supplementary material (Supplement 2).

## Discussion

The present study represents an attempt to evaluate an organizational change that happened during the pandemic of COVID-19 in one of the biggest LHA in Italy with the implementation of the District Operation Centres. The survey was addressed to the main recipient of the creation of the DOCs: GPs and FPs, who were and still are among the first line healthcare figures to face the COVID-19 pandemic [19, 38–40]. Indeed, DOCs were implemented to support GPs and FPs in managing infected patients and their close contacts, especially when frail, and at the same time to serve as a link between them and the public health unit. Medical doctors, GP trainees, nurses and social workers were employed in these DOCs [24].

Definitely, the organization of the Roma 1 LHA represents a first attempt to create synergy among the different components of a complex system as the one regarding primary health care. This structure that was created, notwithstanding all the limits, could be considered an interconnection among the gatekeepers of the National Health Service (GPs and FPs), the public health unit and the local healthcare districts [24]. The fulcrum of this interconnection has been represented by the district, the structure deemed to protect citizens’ health by guaranteeing the essential level of healthcare, through building networks and various levels of integration among stakeholders [41, 42]. The aim with which DOCs were created was to overcome the fragmentation of the primary healthcare system to which health promotion, prevention and healthcare and social interventions belong. According to GPs and FPs of LHA Roma 1, DOCs were useful and reached the intended purpose, even if with differences among the Districts in which the DOCs were implemented. Furthermore, the Roma 1 LHA has planned to maintain the newly devised structures as a fundamental tool for cohesion between all the LHAs and territorial healthcare, while shifting their focus to chronic care and clinical frailty, as already happens in other regions and requested by the most recent national and regional regulations [43–47]. To create proximity health structures, the National Recovery and Resilience Plan (NRRP) has defined structurally the evolution of the INHS while the Ministerial Decree n. 77/2022 defines models and standards of territorial health care [44, 46]. According to these cited regulations, DOCs are placed in the context of a new institutional and organisational structure of the INHS, aimed at building a wider system of transitional care and chronic diseases paths’ coordination [44–47].

COVID-19 pandemic revealed overwhelmingly the fragility of health systems [48] and especially of the primary health care system in several countries, including Italy [14, 15]. However, some lessons can already be learnt such as a full understanding of the role of primary health care in coping with an epidemic (as already mentioned GPs and FPs are the gatekeepers of health system); the need for integration between the different actors at various levels; the support to committed professionals [49] and thus the need to work collaboratively pursuing the same goal [38]; the important and continuous interaction between primary health care and public health [15] and the need to overcome the existing dichotomy also through the integration of public health professionals into the primary health care system [18, 50]; the need for training at all levels [15]. The latter two elements are highlighted in this study by the fact that physicians found the support from DOCs in orienting them about the evolving legislation and the guidance on the certification to be issued. Actually, these lessons resume two of the six lessons for a global primary care response which were summarised by Desborough J et al. from past pandemics: “improve collaboration, communication, and integration between public health and primary care teams and strengthen the primary health care system” [51]. Responsiveness of the health system to sudden threats and health crises has shown to be improved by delivering integrated public health and primary care responses [52], although an international survey addressing primary care experts and investigating their views on their country’s national responses revealed that an effective pandemic response lacked integration of public health and primary care [53].

The effort of the Roma 1 LHA in this pandemic emergency to provide a new service to support medical doctors and consequently patients’ care denotes great leadership in yielding collaboration with other involved stakeholders and having the vision of considering patient-centred healthcare [42, 54].

Another important lesson learned from this pandemic is the need for connections between primary care, public health, and secondary care [15, 50] for example to ensure comprehensive, prompt and adequate care to patients [21]. Unfortunately, the present study did not fully investigate the relationships with secondary care. Only the need to have a connection with specialists of the health district was analysed and that represents a limit of the study. On the other hand, the telephone survey aimed at investigating the relationships of the interviewed doctors with the afferent health district and their possible willingness to actively participate in certain activities to understand whether there are any prerequisites for overcoming the fragmentation of the healthcare system [55].

Among the above-mentioned six key lessons for the global primary care response, we recognize the evaluation of the effectiveness of the intervention, as we did in this attempt. This represents an important aspect to learn from the past in order to better inform policy makers [21]. The methodology with which it was conducted, from the definition of the survey contents to the validation of the same through Delphi, to the sampling and subsequent analysis represents a relevant strength of the study itself. Nevertheless, we recognise that not including FPs in the development of the questionnaire represent a weakness. Although the limitation derived by the cross-sectional design, the rigour followed in the application of the methodology derives on the one hand from the ability of the researchers to apply methodological principles to organisational analysis and on the other hand from the willingness of the LHA to evaluate organisational change, emphasising its strengths and weaknesses, as the company management would like to understand whether to discontinue or maintain, with appropriate changes, these DOCs also after the pandemic. Moreover, it represents one of the few researches analysing the primary care setting in the Italian landscape, especially during or after the COVID-19 pandemic [18, 19, 23].

The vision behind the creation of DOCs is that they will act as a support and glue in primary health care, especially to take charge of chronic, complex, and fragile patients, in cooperation with and considering GPs and FPs’ perspectives [40]. If in the future the management of the LHA decides to maintain DOCs, it would be appropriate -once the emergency is over- to describe their role and their existing and potential interconnections, their functions which could integrate those already carried out by other district services, such as decoding of needs, taking charge, management of transitions between care settings, monitoring of taking care, the provision of integrated social and health services resulting from a multidisciplinary comparison [43]. Therefore, this analysis should be integrated with a mapping of competencies, of structural and technological resources if the LHA management wishes to implement the activities of DOCs, as well as improve the processes of interaction between the nodes of the network to guarantee continuity and integration of care and at the same time managing complexity [43].

## Conclusion

This study aimed to describe how a rapid organisational response, given the spread of the infection, may represent an opportunity through which the primary health care system can be strengthened, modernised, and made patient-centred and resilient for future challenges [14, 56]. Pandemics can lead to develop more resilient systems. In a moment of deep changes for the INHS, the experience of COVID19 needs to be further analysed and “used” for continuously improving the quality and the resilience of the national health system, in particular but not only in the primary health care field. New tools and new models are now under development and - as for the COVID19 in the urban metropolitan context of Rome - these new organizational approaches of primary health care management could be further implemented and disseminated.

### Electronic supplementary material

Below is the link to the electronic supplementary material.

Supplementary Material 1

## Data Availability

The datasets used and/or analysed during the current study are available from the corresponding author on reasonable request.

## Declarations

The study was conducted in accordance with the Declaration of Helsinki. The Lazio Region Ethical Committee approved every part of the study, including the oral informed consent procedure (prot. n. 154/CE Lazio 1).
