# Supplementary material for: The district operation centres in one of the largest local health authorities in Italy to manage COVID-19 surveillance and homecare: first implementation and results of a survey addressed to general practitioners
Source: BMC Health Serv Res. 2023 Nov 7;23:1218. doi: 10.1186/s12913-023-10213-3 (PMC10629134; doi:10.1186/s12913-023-10213-3)
Supplement: Supplementary file 1 — Supplementary Material 1 [file 12913_2023_10213_MOESM1_ESM.docx]

**Supplementary material of the article “The District Operation Centres in one of the largest Local Health Authorities in Italy to manage COVID-19 surveillance and homecare: first implementation and results of a survey addressed to general practitioners.”**

**Supplementary 1. Delphi methodology and questionnaire**

***Delphi methodology***

The Delphi methodology is a widely acknowledged method to agree upon a group opinion or decision by surveying a panel of experts (1,2). Through multiple interactions, experts are asked to express their opinion on the content of the questions of the survey, potentially removing them or adding further ones. The process continues until a common decision is reached (3) and consists of two rounds of discussions at least.

As mentioned in the main text of the article, a minimum of experts to carry out the approach is not defined and for this work we chose to include into the panel: 2 health district directors, 2 public health physicians and 3 General Practitioners (GPs). Family Paediatricians (FPs) were not involved in the development of the questionnaire.

After confirming their availability, they received an email containing the study objectives, instructions on how to fill out the assessment, the questionnaire to assess and the protocol (attachment 1 in supplementary material) describing the assessment criteria and the cut-offs to determine the Validity Content Index (VCI). The purpose of VCI is to assess the validity of the content, which is the ability of the tool to measure the properties of the construct being studied -in our case, the topics the questionnaire deals with. Each expert was asked to validate each question based on general relevance criteria and coherence to the objective of the study through a Likert scale from 1 (strongly disagree -this question should not be included in the questionnaire/it is not relevant) to 5 (strongly agree -the question is relevant and should be included in the questionnaire/it is relevant). The VCI is the rate calculated by dividing the number of experts whose score for each item was equal to or higher than 3 (3, 4 or 5) by the total number of experts involved. Therefore, the VCI value ranges from 0 to 1, that is from 0% to 100%. A VCI higher than or equal to 80% is considered indicative of the item's inclusion in the questionnaire; a value between 70 and 79% is indicative of the need to review the item; whereas a value lower than 70% is suggestive of the item removal. Furthermore, experts were asked to recommend potential changes to the questions as well as to add new ones (4,5). Changes to the questions could concern the text, the removal, and the choice of whether to make the question compulsory or optional.

The first-round questionnaire consisted of 19 scale queries, either open-ended or on a Likert scale, and examined the GPs/FPs’ use of DOCs, their appreciation of both the introduction and the future outlook of DOCs, as related to GPs/FPs’ activity. All the experts replied in the first round and all the questions attained a VCI of 85%. In the second round, the experts evaluated modifications put forward by some of them during the first round. As a result, the survey attached to this supplement comprises 21 questions (open-ended, multiple-choice or based on a Likert scale from 1 to 5, one of the most used (6)); 2 variations of a question were suggested, but none of them achieved a VCI≥80% in the second round, which led to the unmodified question being included in the final survey, as it had a VCI=100% in the first round. In the second round, experts’ participation was 70%, given that 2 physicians did not submit their assessments.

***Attachment 1. Questionnaire***

| **Survey – Role of the District Operation Centres (DOCs) of Local Health Authority Roma 1**  **1. How many years do you practise as GP/FP in your own practice?**   - < 5 years - 5-20 years - 20 years   **2. Are you aware of the existence of the DOCs?**   - Yes, since their institution in April 2020 - Yes, but I found out about them later - I did not know them - Other: ______________________________   **3a. Have you had any contact with the DOC of your District of reference from April 2020 to the present?**   - Yes, occasionally - Yes, constantly - No, never   **3b. Have you had contact with the DOCs of other Districts? If yes, from which District?**   - Yes, ____________________________ - No   **4. If yes, in what way mainly?**   - By phone for __________________ (i.e. asking for info, seeking medical advice, etc…) - By mail for ______________________ - Other: __________________________   **5a. With which healthcare professional figure of the DOC did you interact the most?**   - Medical Doctor - Nurse - Social worker - Other ___________________ - I do not know   **5b. If medical doctor, who was?**   - Director of the District - Doctor of the District - GP trainer hired - GP trainer in internship - Doctor of the Italian Army   **6. If you have been in contact with the DOC of your District of reference, did it support you in:**  **A. Guidance in the evolving legislation**   \|  \| \| 1 \| 2 \| 3 \| 4 \| 5 \|  \| \| --- \| --- \| --- \| --- \| --- \| --- \| --- \| --- \| \|  \| (Not helpful at all) \| \|  \|  \|  \| (Very helpful) \| \|   **B. Managing patient resulted positive to SARS-CoV-2**   \|  \| \| 1 \| 2 \| \| 3 \| 4 \| \| 5 \| \| --- \| --- \| --- \| --- \| --- \| --- \| --- \| --- \| --- \| \| Detection of positive cases \| \|  \|  \| \|  \|  \| \|  \| \| Managing isolation \| \|  \|  \| \|  \|  \| \|  \| \| Managing the end of isolation \| \|  \|  \| \|  \|  \| \|  \| \| Managing cohabitants \| \|  \|  \| \|  \|  \| \|  \| \| Release of certificates (i.e. disease or recovery) or issue “whit” them \| \|  \|  \| \|  \|  \| \|  \| \| Home medical visit request \| \|  \|  \| \|  \|  \| \|  \| \| Request nursing services at home \| \|  \|  \| \|  \|  \| \|  \| \| Request for phone consultation with a specialist \| \|  \|  \| \|  \|  \| \|  \| \|  \| *(Not helpful at all)* \| \| \|  \| \| \| *(Very helpful)* \| \|   **C. Managing COVID-19 close contacts**   \|  \| \| 1 \| 2 \| 3 \| 4 \| 5 \|  \| \| --- \| --- \| --- \| --- \| --- \| --- \| --- \| --- \| \|  \| (Not helpful at all) \| \|  \|  \|  \| (Very helpful) \| \|   **D. Management of a suspected case**   \|  \| \| 1 \| 2 \| 3 \| 4 \| 5 \|  \| \| --- \| --- \| --- \| --- \| --- \| --- \| --- \| --- \| \|  \| (Not helpful at all) \| \|  \|  \|  \| (Very helpful) \| \|   **E. Managing of potential clusters (family members, cohabitants, nursing home, boarding school, college, convent, etc…)**   \|  \| \| 1 \| 2 \| 3 \| 4 \| 5 \|  \| \| --- \| --- \| --- \| --- \| --- \| --- \| --- \| --- \| \|  \| (Not helpful at all) \| \|  \|  \|  \| (Very helpful) \| \|   **F. Relation with the Public Health Department**   \|  \| \| 1 \| 2 \| 3 \| 4 \| 5 \|  \| \| --- \| --- \| --- \| --- \| --- \| --- \| --- \| --- \| \|  \| (Not helpful at all) \| \|  \|  \|  \| (Very helpful) \| \|   **G. Guidance on certifications to be issued**   \|  \| \| 1 \| 2 \| 3 \| 4 \| 5 \|  \| \| --- \| --- \| --- \| --- \| --- \| --- \| --- \| --- \| \|  \| (Not helpful at all) \| \|  \|  \|  \| (Very helpful) \| \|   **H. Use of Regional software (i.e. Lazio Advice)**   \|  \| \| 1 \| 2 \| 3 \| 4 \| 5 \|  \| \| --- \| --- \| --- \| --- \| --- \| --- \| --- \| --- \| \|  \| (Not helpful at all) \| \|  \|  \|  \| (Very helpful) \| \|   **I. USCA* activation for swabs**   \|  \| \| 1 \| 2 \| 3 \| 4 \| 5 \|  \| \| --- \| --- \| --- \| --- \| --- \| --- \| --- \| --- \| \|  \| (Not helpful at all) \| \|  \|  \|  \| (Very helpful) \| \|   *USCA – “special continuity of care units” (Unità Speciali di Continuità Assistenziale)  **L. USCA activation for home medical visits**   \|  \| \| 1 \| 2 \| 3 \| 4 \| 5 \|  \| \| --- \| --- \| --- \| --- \| --- \| --- \| --- \| --- \| \|  \| (Not helpful at all) \| \|  \|  \|  \| (Very helpful) \| \|   *USCA – “special continuity of care units” (Unità Speciali di Continuità Assistenziale)  **M. DOC activation for specialistic consultations or other medical services delivered at home (i.e. blood samples., chest ultrasound)**   \|  \| \| 1 \| 2 \| 3 \| 4 \| 5 \|  \| \| --- \| --- \| --- \| --- \| --- \| --- \| --- \| --- \| \|  \| (Not helpful at all) \| \|  \|  \|  \| (Very helpful) \| \|   Specify any health service delivered at home: ______________________________________  **N. Other (specify): ____________________________________________________________**   \|  \| \| 1 \| 2 \| 3 \| 4 \| 5 \|  \| \| --- \| --- \| --- \| --- \| --- \| --- \| --- \| --- \| \|  \| (Not helpful at all) \| \|  \|  \|  \| (Very helpful) \| \|   **7. Overall, how do you rate the usefulness of the DOC?**   \|  \| \| 1 \| 2 \| 3 \| 4 \| 5 \|  \| \| --- \| --- \| --- \| --- \| --- \| --- \| --- \| --- \| \|  \| (Not helpful at all) \| \|  \|  \|  \| (Very helpful) \| \|   **8. Would you like to give an example of how was it helpful or not helpful?**  ________________________________________________________________________________________________________________________________________________________________________  **9. How do you rate the activity of the DOC during weekends, holidays and pre-holidays in the past months?**   \|  \| \| 1 \| 2 \| 3 \| 4 \| 5 \|  \| \| --- \| --- \| --- \| --- \| --- \| --- \| --- \| --- \| \|  \| (Not helpful at all) \| \|  \|  \|  \| (Very helpful) \| \|   **10. BEFORE COVID-19 pandemic, what was your availability to your patients on weekends, pre-holidays and holidays?**   - Not available (there is the Continuity of Care Service) - Partially available - Very available   **11. DURING COVID-19 pandemic, what was your availability to your patients on weekends, pre-holidays and holidays?**   - Not available (there is the Continuity of Care Service) - Partially available - Very available   **12. If you answered “Partially available” or “Very available” would you have appreciated a service like the DOC during weekends and holidays?**   \|  \| 1 \| 2 \| 3 \| 4 \| 5 \|  \| \| --- \| --- \| --- \| --- \| --- \| --- \| --- \| \| (Not at all) \| \|  \|  \|  \| (A lot) \| \|   **13. BEFORE COVID-19 pandemic, would you have liked a service of the LHA on the model of the current DOC in order to support you practice?**   \|  \| 1 \| 2 \| 3 \| 4 \| 5 \|  \| \| --- \| --- \| --- \| --- \| --- \| --- \| --- \| \| (Not at all) \| \|  \|  \|  \| (A lot) \| \|   **14. BEFORE COVID-19 pandemic had you had any contact with District’s services? (i.e. home care, prosthetic service, disability/frailty service…)**   - Yes - Not - Sometimes   **15. If you answered yes or sometimes, to which ones?**  ________________________________________________________________________________________________________________________________________________________________________  **16. BEFORE COVID-19 pandemic, did you actively participate in activities organised by the District? (i.e. committees, audit, meeting for the unities of primary care or continuity of care services during weekends, meetings with external stakeholders – associations, Municipalities… – etc.)**   - Yes - Not - Sometimes   **17. If you answered yes or sometimes, to which ones?**  ________________________________________________________________________________________________________________________________________________________________________  **18. Would you recommend to other Local Health Authorities a service to support GPs/FPs on the model of DOC?**   \|  \| 1 \| 2 \| 3 \| 4 \| 5 \|  \| \| --- \| --- \| --- \| --- \| --- \| --- \| --- \| \| (Not at all) \| \|  \|  \|  \| (A lot) \| \|   **19. What do you think could be improved or changed about the current organisation of the DOC in the relation between the DOC and GPs/FPs? Do you have any suggestions for future developments? (several answers possible)**   - Hours availability - On call - Dedicated phone number/mail - Relation with the Public Health Department - Proactivity of the LHA - Relation with specialists - Other _______________________________________   **20. Let’s look beyond COVID-19 pandemic: do you think that a service modelled on the DOC could be useful in the future?**  **A. For managing frail patients**   \|  \| 1 \| 2 \| 3 \| 4 \| 5 \|  \| \| --- \| --- \| --- \| --- \| --- \| --- \| --- \| \| (I don’t think it’s useful) \| \|  \|  \|  \| (I think it’s very useful) \| \|   **B. For the relation with specialists**   \|  \| 1 \| 2 \| 3 \| 4 \| 5 \|  \| \| --- \| --- \| --- \| --- \| --- \| --- \| --- \| \| (I don’t think it’s useful) \| \|  \|  \|  \| (I think it’s very useful) \| \|   **C. Counselling to patients (i.e. nurse counselling)**   \|  \| 1 \| 2 \| 3 \| 4 \| 5 \|  \| \| --- \| --- \| --- \| --- \| --- \| --- \| --- \| \| (I don’t think it’s useful) \| \|  \|  \|  \| (I think it’s very useful) \| \|   **D. Telemedicine services and support to the use of Regional software**   \|  \| 1 \| 2 \| 3 \| 4 \| 5 \|  \| \| --- \| --- \| --- \| --- \| --- \| --- \| --- \| \| (I don’t think it’s useful) \| \|  \|  \|  \| (I think it’s very useful) \| \|   **E. Other (specify): _______________________________________________**   \|  \| 1 \| 2 \| 3 \| 4 \| 5 \|  \| \| --- \| --- \| --- \| --- \| --- \| --- \| --- \| \| (I don’t think it’s useful) \| \|  \|  \|  \| (I think it’s very useful) \| \|   **21. Would you like to be involved more in District activities in the future?**  **A. Design and implementation of diagnostic and therapeutic care pathways**   \|  \| 1 \| 2 \| 3 \| 4 \| 5 \|  \| \| --- \| --- \| --- \| --- \| --- \| --- \| --- \| \| (Not at all) \| \|  \|  \|  \| (A lot) \| \|   **B. Health promotion activities (i.e. counselling, population meetings…)**   \|  \| 1 \| 2 \| 3 \| 4 \| 5 \|  \| \| --- \| --- \| --- \| --- \| --- \| --- \| --- \| \| (Not at all) \| \|  \|  \|  \| (A lot) \| \|   **C. Primary and secondary health prevention activities (i.e. vaccinations, screenings…)**   \|  \| 1 \| 2 \| 3 \| 4 \| 5 \|  \| \| --- \| --- \| --- \| --- \| --- \| --- \| --- \| \| (Not at all) \| \|  \|  \|  \| (A lot) \| \|   **D. Other (specify): ___________________________________________________**   \|  \| 1 \| 2 \| 3 \| 4 \| 5 \|  \| \| --- \| --- \| --- \| --- \| --- \| --- \| --- \| \| (Not at all) \| \|  \|  \|  \| (A lot) \| \| |
| --- | --- | --- | --- | --- | --- | --- | --- | --- | --- | --- | --- | --- | --- | --- | --- | --- | --- | --- | --- | --- | --- | --- | --- | --- | --- | --- | --- | --- | --- | --- | --- | --- | --- | --- | --- | --- | --- | --- | --- | --- | --- | --- | --- | --- | --- | --- | --- | --- | --- | --- | --- | --- | --- | --- | --- | --- | --- | --- | --- | --- | --- | --- | --- | --- | --- | --- | --- | --- | --- | --- | --- | --- | --- | --- | --- | --- | --- | --- | --- | --- | --- | --- | --- | --- | --- | --- | --- | --- | --- | --- | --- | --- | --- | --- | --- | --- | --- | --- | --- | --- | --- | --- | --- | --- | --- | --- | --- | --- | --- | --- | --- | --- | --- | --- | --- | --- | --- | --- | --- | --- | --- | --- | --- | --- | --- | --- | --- | --- | --- | --- | --- | --- | --- | --- | --- | --- | --- | --- | --- | --- | --- | --- | --- | --- | --- | --- | --- | --- | --- | --- | --- | --- | --- | --- | --- | --- | --- | --- | --- | --- | --- | --- | --- | --- | --- | --- | --- | --- | --- | --- | --- | --- | --- | --- | --- | --- | --- | --- | --- | --- | --- | --- | --- | --- | --- | --- | --- | --- | --- | --- | --- | --- | --- | --- | --- | --- | --- | --- | --- | --- | --- | --- | --- | --- | --- | --- | --- | --- | --- | --- | --- | --- | --- | --- | --- | --- | --- | --- | --- | --- | --- | --- | --- | --- | --- | --- | --- | --- | --- | --- | --- | --- | --- | --- | --- | --- | --- | --- | --- | --- | --- | --- | --- | --- | --- | --- | --- | --- | --- | --- | --- | --- | --- | --- | --- | --- | --- | --- | --- | --- | --- | --- | --- | --- | --- | --- | --- | --- | --- | --- | --- | --- | --- | --- | --- | --- | --- | --- | --- | --- | --- | --- | --- | --- | --- | --- | --- | --- | --- | --- | --- | --- | --- | --- | --- | --- | --- | --- | --- | --- | --- | --- | --- | --- | --- | --- | --- | --- | --- | --- | --- | --- | --- | --- | --- | --- | --- | --- | --- | --- | --- | --- | --- | --- | --- | --- | --- | --- | --- | --- | --- | --- | --- | --- | --- | --- | --- | --- | --- | --- | --- | --- | --- | --- | --- | --- | --- | --- | --- | --- | --- | --- | --- | --- | --- | --- | --- | --- | --- | --- | --- | --- | --- | --- | --- | --- | --- | --- | --- | --- | --- | --- | --- | --- | --- | --- | --- | --- | --- | --- | --- | --- | --- | --- | --- | --- | --- | --- | --- | --- | --- | --- | --- | --- | --- | --- | --- | --- | --- | --- | --- | --- | --- | --- | --- | --- | --- | --- | --- | --- | --- | --- | --- | --- | --- | --- | --- | --- | --- | --- | --- | --- | --- | --- | --- | --- | --- | --- | --- | --- | --- | --- | --- | --- | --- | --- | --- | --- | --- | --- | --- | --- | --- | --- | --- | --- | --- | --- | --- | --- | --- | --- | --- | --- | --- | --- | --- | --- | --- | --- | --- | --- | --- | --- | --- | --- |

**Supplement 2. Sample size determination, Survey Results and Geospatial References**

**Sample size determination**

The district sample size used (in grey in table S2.1) was close to 20% (n=197 out of 984) of the whole GPs/FPs population of the Roma 1 LHA and, without considering the GPs/FPs’ district distribution, it would have provided information with an 80% CL and a 5% MOE; conversely, a 212 subjects sample size would achieve a 90% CL and a 5% MOE. For this reason, to further strengthen the information obtained from the survey, we have decided to continue with the interviews even after reaching the predefined minimum goal.

**Table S2.1**. GPs and FPs population per District and sample size determination.

| **District** | **GPs/FPs population** | **80% CL and 10% MOE** | **90% CL and 10% MOE** | **95% CL and 10% MOE** | **80% CL and 5% MOE** | **90% CL and 5% MOE** | **95% CL and 5% MOE** |
| --- | --- | --- | --- | --- | --- | --- | --- |
| 1 | 195 | 34 | 50 | 64 | 89 | 113 | 129 |
| 2 | 190 | 34 | 50 | 64 | 88 | 112 | 127 |
| 3 | 194 | 34 | 50 | 64 | 89 | 113 | 129 |
| 13 | 125 | 31 | 44 | 54 | 71 | 85 | 94 |
| 14 | 162 | 33 | 48 | 60 | 82 | 101 | 114 |
| 15 | 118 | 30 | 43 | 53 | 69 | 82 | 90 |
| Total | 984 | 196 | 285 | 359 | 488 | 606 | 683 |

**Survey results**

Overall, between December 2020 and January 2021, 215 subjects (corresponding to 21.9% of all GPs/FPs of LHA) were interviewed. The sample target was reached both for the health districts (80% CL and 10% MOE) and for the whole LHA (90% CL and 5% MOE) level. The district distribution of interviewed subjects as compared to the district sample size is described in Table S2.2.

**Table S2.2.** Number of GPs/FPs interviewed per District.

| **District (DOC)** | **Total number of GPs/FPs per District (N)** | **GPs/FPs**  **sample size**  **(CL 80%,**  **MOE 10%)** | **GPs/FPs interviewed (n)** | **% of GPs/FPs interviewed per District (n/N)** | **% of GPs/FPs interviewed / total GPs/FPs interviewed** |
| --- | --- | --- | --- | --- | --- |
| 1 | 195 | 34 | 36 | 18.5 | 16.7 |
| 2 | 190 | 34 | 39 | 20.5 | 18.1 |
| 3 | 194 | 34 | 36 | 18.6 | 16.7 |
| 13 | 125 | 31 | 36 | 28.8 | 16.7 |
| 14 | 162 | 33 | 36 | 22.2 | 16.7 |
| 15 | 118 | 30 | 32 | 27.1 | 14.9 |
| Total | 984 | 196 | 215* | 21.9 | 100 |

*212 was the sample size necessary to reach 90% CL e 5% MOE considering the entire GPs/FPs population of LHA Roma 1, but not their district distribution.

Nearly half of the interviewed sample (56.7%) had a proper medical office as GP/FP for more than 20 years, 28.8% in a range from 5 to 20 years, and 14.4% for less than 5 years (data not shown).

Table S2.3 shows the degree of knowledge of the DOCs by GPs/FPs (question 2). 84.6% of the interviewed sample stated that they had known about DOCs since their institution (April 2020), whereas the remaining 14.9% found out about them later on. The district that had the widest spread of information about DOCs institutions was District 13, where 91.7% of the GPs/FPs interviewed knew of them ever since their establishment, although the difference with the other districts was not statistically significant (p = 0.42).

**Table S2.3.** GPs/FPs answers to question asking the degree of knowledge of the DOCs, grouped by health district.

| **Knowledge of Roma 1 LHA DOCs existence?** | **Health District** | | | | | | **Total** |
| --- | --- | --- | --- | --- | --- | --- | --- |
|  | **1** | **2** | **3** | **13** | **14** | **15** |  |
| No, I did not know about them | 1 (2.8%) | 0 (0%) | 0 (0%) | 0 (0%) | 0 (0%) | 0 (0%) | 1 (0.5%) |
| Yes, but I found out about them later on | 6 (16.7%) | 10 (25.6%) | 5 (13.9%) | 3 (8.3%) | 4 (11.1%) | 4 (12.5%) | 32 (14.9%) |
| Yes, since their institution | 29 (80.5%) | 29 (74.4%) | 31 (86.1%) | 33 (91.7%) | 32 (88.9%) | 28 (87.5%) | 182 (84.6%) |
| Total | 36 (100%) | 39 (100%) | 36 (100%) | 36 (100%) | 36 (100%) | 32 (100%) | 215 (100%) |

52.1% of the GPs/FPs interviewed reported that they had frequent contacts with their DOC, while 46.5% had occasional contacts. Slightly more than half stated that they did not have any contact with DOCs of other districts, 49.5% asserted that they had contacts with other DOCs (data not shown).

When analysing the frequency of replies to question concerning the overall usefulness, the median score of the 5-point Likert scale was 4 (Q1-Q3: 3-5). 55.4% of respondents found that DOCs were useful during the pandemic (118 out of 213 scored 4 to 5 on a 5-point Liker scale) although no statistically significant differences were detected. The median, Q1, Q3, the mean and standard deviation of replies to the question are listed in Table S2.4 with the comparison among each district and all the others considered as one district.

For question assessing the usefulness of DOCs, there were no statistically significant differences between the districts and all the others considered as one, although the doctors of Districts 3 and 13 seem to have given answers that differed from those of the doctors from other Districts (no significant statistical differences).

**Table S2.4**. Replies to question “Overall, how do you rate the DOC usefulness?” divided by district. N. 2 answers missing (both from physicians of District 1).

|  | **District** | **N.** | **Median** | **Q1-Q3** | **Mean** | **SD** | **p-value** |
| --- | --- | --- | --- | --- | --- | --- | --- |
| **DOC usefulness** | **1** | 34 | 4 | 3-5 | 3.70 | 1.36 | 0.49 |
|  | **2** | 39 | 3 | 3-5 | 3.53 | 1.21 | 0.56 |
|  | **3** | 36 | 3 | 3-4 | 3.28 | 1.26 | 0.06 |
|  | **13** | 36 | 4 | 3-5 | 3.86 | 1.36 | 0.09 |
|  | **14** | 36 | 4 | 3-5 | 3.75 | 1.10 | 0.57 |
|  | **15** | 32 | 4 | 3-4 | 3.53 | 1.14 | 0.62 |
|  | **Total** | **213** | **4** | **3-5** | **3.61** | **1.24** |  |

Table S2.5, instead, reports the median, Q1 and Q3, the mean and standard deviation for each of the options for the question concerned about the utility of the DOC in each specific activity in which the DOC was involved.

**Table S2.5.** Replies to question “If you had contact with Your DOC: it was useful in…”. N. 1 answer missing (from physician of District 1); in replies to the question “G. Support on certificates to issue” n. 2 answers missing (from one physician of District 1 and one from District 3).

|  | **District** | **N.** | **Median** | **Q1-Q3** | **Mean** | **SD** | ***p value*** |
| --- | --- | --- | --- | --- | --- | --- | --- |
| **A. Support on**  **legislation changes** | **1** | 35 | 4 | 3-5 | 3.54 | 1.40 | 0.46 |
|  | **2** | 39 | 3 | 1-4 | 2.90 | 1.50 | 0.02* |
|  | **3** | 36 | 3.5 | 3-5 | 3.56 | 1.16 | 0.68 |
|  | **13** | 36 | 4 | 3-5 | 3.56 | 1.40 | 0.41 |
|  | **14** | 36 | 4 | 2-5 | 3.39 | 1.40 | 0.95 |
|  | **15** | 32 | 4 | 3-4 | 3.59 | 1.10 | 0.56 |
|  | **Total** | **214** | **4** | **2-5** | **3.41** | **1.35** |  |
| **B. Management of Covid-19 positive cases:** |  |  |  |  |  |  |  |
| *1. Case detection* | **1** | 35 | 1 | 1-3 | 2.06 | 1.51 | 0.36 |
|  | **2** | 39 | 2 | 1-3 | 2.31 | 1.52 | 0.73 |
|  | **3** | 36 | 1 | 1-2.5 | 1.89 | 1.37 | 0.10 |
|  | **13** | 36 | 1 | 1-4 | 2.28 | 1.65 | 0.94 |
|  | **14** | 36 | 2.5 | 1-4 | 2.58 | 1.48 | 0.09 |
|  | **15** | 32 | 2 | 1-4 | 2.38 | 1.54 | 0.57 |
|  | **Total** | **214** | **1** | **1-4** | **2.25** | **1.51** |  |
| *2. Isolation management* | **1** | 35 | 3 | 1-4 | 2.71 | 1.54 | 0.55 |
|  | **2** | 39 | 3 | 1-4 | 2.67 | 1.50 | 0.64 |
|  | **3** | 36 | 1 | 1-3 | 2.00 | 1.41 | 0.01* |
|  | **13** | 36 | 3 | 1-5 | 2.97 | 1.65 | 0.09 |
|  | **14** | 36 | 2 | 1-4 | 2.44 | 1.46 | 0.63 |
|  | **15** | 32 | 3 | 1-4 | 2.69 | 1.53 | 0.69 |
|  | **Total** | **214** | **2** | **1-4** | **2.58** | **1.53** |  |
| *3. Halt the management of isolation* | **1** | 35 | 2 | 1-4 | 2.54 | 1.54 | 0.91 |
|  | **2** | 39 | 3 | 1-3 | 2.51 | 1.41 | 0.97 |
|  | **3** | 36 | 1 | 1-3 | 2.03 | 1.42 | 0.02* |
|  | **13** | 36 | 3 | 1-5 | 3.03 | 1.68 | 0.06 |
|  | **14** | 36 | 2 | 1-4 | 2.44 | 1.49 | 0.75 |
|  | **15** | 32 | 3 | 1-4 | 2.78 | 1.64 | 0.44 |
|  | **Total** | **214** | **2** | **1-4** | **2.55** | **1.54** |  |
| *4. Cohabitees’ management* | **1** | 35 | 3 | 1-4 | 2.74 | 1.58 | 0.19 |
|  | **2** | 39 | 3 | 1-3 | 2.54 | 1.33 | 0.47 |
|  | **3** | 36 | 1 | 1-3 | 2.00 | 1.33 | 0.04* |
|  | **13** | 36 | 2.5 | 1-4 | 2.61 | 1.59 | 0.47 |
|  | **14** | 36 | 1.5 | 1-3 | 2.03 | 1.23 | 0.09 |
|  | **15** | 32 | 3 | 1-4 | 2.66 | 1.47 | 0.32 |
|  | **Total** | **214** | **2** | **1-4** | **2.43** | **1.44** |  |
| *5. Issue of certificates* | **1** | 35 | 1 | 1-3 | 2.00 | 1.28 | 0.85 |
|  | **2** | 39 | 1 | 1-3 | 2.05 | 1.41 | 0.75 |
|  | **3** | 36 | 1 | 1-2.5 | 1.78 | 1.20 | 0.13 |
|  | **13** | 36 | 1.5 | 1-4 | 2.39 | 1.59 | 0.29 |
|  | **14** | 36 | 2 | 1-4 | 2.39 | 1.52 | 0.23 |
|  | **15** | 32 | 1 | 1-3 | 2.00 | 1.32 | 0.80 |
|  | **Total** | **214** | **1** | **1-3** | **2.10** | **1.39** |  |
| *6. Request for a medical home visit* | **1** | 35 | 1 | 1-4 | 2.23 | 1.65 | 0.89 |
|  | **2** | 39 | 1 | 1-4 | 2.13 | 1.63 | 0.72 |
|  | **3** | 36 | 1 | 1-2 | 1.78 | 1.44 | 0.05* |
|  | **13** | 36 | 1 | 1-3 | 1.94 | 1.60 | 0.16 |
|  | **14** | 36 | 2 | 1-4.5 | 2.69 | 1.74 | 0.05* |
|  | **15** | 32 | 3 | 1-4 | 2.62 | 1.54 | 0.08 |
|  | **Total** | **214** | **1** | **1-4** | **2.22** | **1.62** |  |
| *7. Request for nurse services at home* | **1** | 35 | 1 | 1-3 | 1.97 | 1.62 | 0.59 |
|  | **2** | 39 | 1 | 1-3 | 1.92 | 1.51 | 0.42 |
|  | **3** | 36 | 1 | 1-2 | 1.69 | 1.17 | 0.25 |
|  | **13** | 36 | 1 | 1-4 | 2.05 | 1.64 | 0.79 |
|  | **14** | 36 | 1.5 | 1-4 | 2.47 | 1.68 | 0.08 |
|  | **15** | 32 | 1 | 1-4 | 2.31 | 1.55 | 0.29 |
|  | **Total** | **214** | **1** | **1-3** | **2.07** | **1.54** |  |
| *8. Request for telephone consultation with a specialist* | **1** | 35 | 1 | 1-1 | 1.63 | 1.33 | 0.60 |
|  | **2** | 39 | 1 | 1-1 | 1.56 | 1.29 | 0.86 |
|  | **3** | 36 | 1 | 1-1 | 1.39 | 0.96 | 0.78 |
|  | **13** | 36 | 1 | 1-1 | 1.56 | 1.23 | 0.48 |
|  | **14** | 36 | 1 | 1-1 | 1.28 | 0.88 | 0.24 |
|  | **15** | 32 | 1 | 1-1 | 1.43 | 1.01 | 0.95 |
|  | **Total** | **214** | **1** | **1-1** | **1.48** | **1.13** |  |
| **C. Covid-19 case contacts’ management** | **1** | 35 | 2 | 1-4 | 2.57 | 1.61 | 0.57 |
|  | **2** | 39 | 2 | 1-3 | 2.33 | 1.39 | 0.85 |
|  | **3** | 36 | 2 | 1-3 | 2.19 | 1.39 | 0.46 |
|  | **13** | 36 | 3 | 1-4 | 2.69 | 1.69 | 0.35 |
|  | **14** | 36 | 1 | 1-3.5 | 2.11 | 1.45 | 0.16 |
|  | **15** | 32 | 3 | 1-4 | 2.66 | 1.58 | 0.38 |
|  | **Total** | **214** | **2** | **1-4** | **2.42** | **1.52** |  |
| **D. Suspected patients’ management** | **1** | 35 | 1 | 1-4 | 2.14 | 1.63 | 0.56 |
|  | **2** | 39 | 2 | 1-4 | 2.41 | 1.44 | 0.21 |
|  | **3** | 36 | 1 | 1-4 | 2.17 | 1.44 | 0.85 |
|  | **13** | 36 | 1.5 | 1-4 | 2.44 | 1.65 | 0.37 |
|  | **14** | 36 | 1 | 1-3 | 1.78 | 1.24 | 0.06 |
|  | **15** | 32 | 2 | 1-3 | 2.28 | 1.42 | 0.63 |
|  | **Total** | **214** | **1** | **1-3** | **2.20** | **1.48** |  |
| **E. Clusters’ management** *(in boarding school, college, convent, nursing home, etc.)* | **1** | 35 | 1 | 1-4 | 2.29 | 1.67 | 0.70 |
|  | **2** | 39 | 1 | 1-3 | 2.00 | 1.43 | 0.16 |
|  | **3** | 36 | 2 | 1-4 | 2.50 | 1.50 | 0.43 |
|  | **13** | 36 | 3 | 1-5 | 2.91 | 1.75 | 0.03* |
|  | **14** | 36 | 2.5 | 1-4 | 2.67 | 1.64 | 0.18 |
|  | **15** | 32 | 1 | 1-2.5 | 1.69 | 1.20 | 0.01* |
|  | **Total** | **214** | **1** | **1-4** | **2.35** | **1.58** |  |
| **F. Interaction with Hygiene and Public Health Unit** | **1** | 35 | 2 | 1-5 | 2.69 | 1.83 | 0.95 |
|  | **2** | 39 | 3 | 1-4 | 2.59 | 1.50 | 0.82 |
|  | **3** | 36 | 2 | 1-4 | 2.64 | 1.64 | 0.91 |
|  | **13** | 36 | 2 | 1-4,5 | 2.67 | 1.69 | 0.98 |
|  | **14** | 36 | 3 | 1-5 | 2.86 | 1.69 | 0.41 |
|  | **15** | 32 | 2 | 1-3 | 2.43 | 1.34 | 0.63 |
|  | **Total** | **214** | **2** | **1-4** | **2.65** | **1.61** |  |
| **G. Support on certificates to issue** | **1** | 35 | 3 | 1-4 | 2.66 | 1.43 | 0.73 |
|  | **2** | 39 | 2 | 1-3 | 2.33 | 1.46 | 0.06 |
|  | **3** | 35 | 4 | 1-4 | 2.97 | 1.62 | 0.34 |
|  | **13** | 36 | 3.5 | 1-5 | 3.17 | 1.59 | 0.07 |
|  | **14** | 36 | 3 | 1-4 | 2.78 | 1.62 | 0.87 |
|  | **15** | 32 | 3 | 1-4 | 2.59 | 1.36 | 0.53 |
|  | **Total** | **213** | **3** | **1-4** | **2.75** | **1.53** |  |
| **H. Use of regional digital platforms** | **1** | 35 | 1 | 1-3 | 1.91 | 1.54 | 0.84 |
|  | **2** | 39 | 1 | 1-2 | 1.74 | 1.31 | 0.40 |
|  | **3** | 36 | 1 | 1-2 | 1.56 | 1.05 | 0.20 |
|  | **13** | 36 | 1 | 1-3.5 | 2.19 | 1.60 | 0.20 |
|  | **14** | 36 | 1 | 1-3 | 1.80 | 1.19 | 0.84 |
|  | **15** | 32 | 1,5 | 1-2 | 1.78 | 1.00 | 0.37 |
|  | **Total** | **214** | **1** | **1-2** | **1.83** | **1.30** |  |
| **I. Activation of USCA to perform COVID-19 tests** | **1** | 35 | 4 | 1-5 | 3.11 | 1.79 | 0.97 |
|  | **2** | 39 | 4 | 2-5 | 3.43 | 1.59 | 0.18 |
|  | **3** | 36 | 3 | 1-4 | 2.61 | 1.54 | 0.03* |
|  | **13** | 36 | 3 | 1-5 | 2.97 | 1.87 | 0.65 |
|  | **14** | 36 | 4 | 2.5-5 | 3.64 | 1.51 | 0.04* |
|  | **15** | 32 | 3 | 1.5-4 | 2.88 | 1.50 | 0.35 |
|  | **Total** | **214** | **3** | **1-5** | **3.11** | **1.66** |  |
| **L. Activation of USCA to perform home visits** | **1** | 35 | 1 | 1-4 | 2.09 | 1.69 | 0.20 |
|  | **2** | 39 | 1 | 1-4 | 2.41 | 1.70 | 0.69 |
|  | **3** | 36 | 1 | 1-3 | 1.97 | 1.40 | 0.18 |
|  | **13** | 36 | 1 | 1-4 | 2.30 | 1.73 | 0.69 |
|  | **14** | 36 | 2 | 1-5 | 2.78 | 1.79 | 0.10 |
|  | **15** | 32 | 2 | 1-4 | 2.53 | 1.57 | 0.30 |
|  | **Total** | **214** | **1** | **1-4** | **2.35** | **1.66** |  |
| **M. Specialized consultations visits and other services delivered at home** | **1** | 35 | 1 | 1-1 | 1.49 | 1.17 | 0.69 |
|  | **2** | 39 | 1 | 1-1 | 1.62 | 1.33 | 0.85 |
|  | **3** | 36 | 1 | 1-1 | 1.44 | 1.03 | 0.82 |
|  | **13** | 36 | 1 | 1-1 | 1.64 | 1.40 | 0.93 |
|  | **14** | 36 | 1 | 1-1 | 1.53 | 1.18 | 0.92 |
|  | **15** | 32 | 1 | 1-1.5 | 1.5 | 0.98 | 0.63 |
|  | **Total** | **214** | **1** | **1-1** | **1.54** | **1.18** |  |

Note: The table includes the number of replies per each question (N), median, IQR i.e. the 25^ (Q1) and 75^ (Q3) percentile, mean and standard deviation (SD). Total values and values divided by district are reported for each question. In each question, the lowest and highest values permitted for the answer were the upper and lower limit of the range that is, respectively, 1 and 5. The Two-sample Wilcoxon rank-sum (Mann-Whitney U) test was used to compare each district’s replies versus all the other Districts considered (e.g., District 1 vs Districts 2, 3, 13, 14 and 15 all together).

* means a p value ≤ 0.05

About the process "management of the positive case", the DOC 3 presented lower values compared to those of the other DOCs. These were referring to the sub-items investigating "management of the isolation of the positive case" (p=0.01), "Halt the management of isolation" (p=0.02), “request for a medical home visit” (p=0.05) and the "activation of USCAR to perform COVID-19 tests” (p=0.03). Also, within the same area but concerning the home visits in people who resulted positive to the SARS-CoV-2, the DOC 3 was less supportive while DOC 14 was more active when compared with all the other districts (statistically significant differences). Considering the management of close contacts living in a household, there was a statistically significant difference between DOC 3 and the others, so that support for this function was worse than the other DOCs.

Concerning cluster management, statistically significant differences were found when comparing DOC 13 and DOC 15 versus all others: DOC 13 appeared to have been able to provide better support to the activity of GPs and FPs than the others, while District 15 had the worst performance in this.

Regarding the item "USCAR activation for the execution of swabs", a statistically significant difference was observed between the DOC 3 performance, which appears worse than that of the other districts, and the performance of DOC 14, which appears better than all the other districts.

Answers to questions investigated the availability of GPs/FPs on weekends, on public holidays and pre-holidays showed that 50.9% of GPs/FPs were very available to the patients before the pandemic, and this percentage reached 93.9% during the pandemic.

Conversely, the evaluation of the activity of the DOCs on Saturdays, Sundays, public holidays and days before public holidays was assessed by most of the respondents (n. 109) with a rating of 1 or 2 on a Likert scale.

Among the GPs/FPs who answered “partially available” or “very available” on weekends, on public holidays and pre-holidays 76.9% of them liked that the DOCs were also open on Saturdays, Sundays, and evenings: precisely, 51.9% scoring the highest rating (level 5 on a 5-point Likert scale), while 25% chose level 4. Moreover, most of the physicians interviewed stated that they would have appreciated work support like the one provided by the DOCs also before the pandemic (58.9% scored 4 to 5 on Likert scale). Indeed, investigating contacts with district services before the pandemic, 53.3% of the doctors interviewed stated that they had had frequent contact with services, while for 19.2% the contact was sporadic. Most of these contacts were with the home care service (percentage not available, 116 responses).

The percentage of doctors participating in activities or functions organised by the district (such as audits, commissions and meetings with other stakeholders) was 35.1% for those who constantly participated in these activities/functions and 32.3% for those who attended the activities only sometimes, while 32.7% of the respondents declared that they never took part in the above-mentioned activities/functions.

Most of the doctors interviewed stated that they would recommend to other local health authorities a service based on the model of the DOC to support of GPs/PFs (over 78.5% scored 4 to 5 on Likert scale).

When asked what should be improved or changed about the DOCs (n.79 respondents), the majority affirmed that on-call scheduling should be improved, with a dedicated number and email. Improvements are needed also in the relationship with the specialists (percentages higher than 60%), in the hourly availability and in the relationship with the public health unit (percentages close to 60%). Moreover, according to GPs/FPs, the DOC could be useful for the management of frail patients, contact with specialists, counselling to patients and support in the use of telemedicine and regional applications (Table S2.6). Similarly, although with slightly lower response values, local doctors would like to be involved in some health district activities, such as the design and implementation of the diagnostic-therapeutic care pathways, health promotion activities and primary and secondary prevention activities (Table S2.7).

**Table S2.6.** Replies to question “Looking beyond the pandemic: do you maintain that a DOC-based service could be useful in the future?”. N. 1 answer missing (from physician of District 15); in replies to the question “D. For telehealth services and regional software support” n. 3 answers missing (from physicians of District 3 and District 15, respectively one and two physicians each).

|  | **District** | **N.** | **Median** | **Q1-Q3** | **Mean** | **SD** |
| --- | --- | --- | --- | --- | --- | --- |
| **A. To manage frail patients** | **1** | 36 | 5 | 4-5 | 4.39 | 0.99 |
|  | **2** | 39 | 5 | 4-5 | 4.49 | 0.85 |
|  | **3** | 36 | 5 | 4-5 | 4.55 | 0.81 |
|  | **13** | 36 | 5 | 5-5 | 4.67 | 0.89 |
|  | **14** | 36 | 5 | 4-5 | 4.36 | 0.90 |
|  | **15** | 31 | 5 | 4-5 | 4.12 | 1.26 |
|  | **Total** | **214** | **5** | **4-5** | **4.44** | **0.96** |
| **B. For contacting medical specialists** | **1** | 36 | 5 | 4-5 | 4.47 | 0.97 |
|  | **2** | 39 | 5 | 4-5 | 4.23 | 1.16 |
|  | **3** | 36 | 5 | 4-5 | 4.22 | 1.20 |
|  | **13** | 36 | 5 | 5-5 | 4.69 | 0.67 |
|  | **14** | 36 | 5 | 4-5 | 4.41 | 1.05 |
|  | **15** | 31 | 4 | 3-5 | 3.70 | 1.35 |
|  | **Total** | **214** | **5** | **4-5** | **4.30** | **1.10** |
| **C. For patient counselling** | **1** | 36 | 5 | 4-5 | 4.17 | 1.20 |
|  | **2** | 39 | 5 | 4-5 | 4.43 | 0.91 |
|  | **3** | 36 | 4 | 3-5 | 3.72 | 1.37 |
|  | **13** | 36 | 5 | 4-5 | 4.39 | 1.02 |
|  | **14** | 36 | 5 | 3-5 | 3.94 | 1.30 |
|  | **15** | 31 | 4 | 3-5 | 3.77 | 1.28 |
|  | **Total** | **214** | **5** | **4-5** | **4.08** | **1.20** |
| **D. For telehealth services and regional software support** | **1** | 36 | 4.5 | 3-5 | 3.97 | 1.30 |
|  | **2** | 39 | 4 | 3-5 | 3.77 | 1.39 |
|  | **3** | 35 | 4 | 3-5 | 3.85 | 1.11 |
|  | **13** | 36 | 4 | 3-5 | 3.94 | 1.26 |
|  | **14** | 36 | 4 | 2.5-5 | 3.42 | 1.44 |
|  | **15** | 30 | 4 | 2-4 | 3.53 | 1.20 |
|  | **Total** | **212** | **4** | **3-5** | **3.75** | **1.29** |

Note: the number of replies to each question, median, Q1-Q3 and the mean and SD are reported. The total values and values divided by district are reported in the table. In all questions, the maximum and minimum values correspond to the upper and lower limit of the Likert scale that is 5 and 1.

**Table S2.7.** Replies to question “Would you like to be more involved in district activities in the future?”. N. 2 answers missing (1 from physicians of District 3 and District 15, respectively).

|  | **District** | **N.** | **Median** | **Q1-Q3** | **Mean** | **SD** |
| --- | --- | --- | --- | --- | --- | --- |
| **A. Design and implementation of clinical pathways** | **1** | 36 | 4 | 3-5 | 3.83 | 1.42 |
|  | **2** | 39 | 4 | 3-5 | 3.62 | 1.37 |
|  | **3** | 35 | 4 | 1-4 | 3.17 | 1.56 |
|  | **13** | 36 | 3.5 | 2-5 | 3.39 | 1.54 |
|  | **14** | 36 | 3.5 | 2-4 | 3.10 | 1.46 |
|  | **15** | 31 | 3 | 1-4 | 2.80 | 1.60 |
|  | **Total** | **213** | **4** | **2-5** | **3.33** | **1.50** |
| **B. Health promotion activities** | **1** | 36 | 4 | 3-5 | 3.92 | 1.30 |
|  | **2** | 39 | 3 | 1-5 | 3.08 | 1.61 |
|  | **3** | 35 | 3 | 1-4 | 2.89 | 1.53 |
|  | **13** | 36 | 4 | 1.5-5 | 3.19 | 1.58 |
|  | **14** | 36 | 2.5 | 2-5 | 3.00 | 1.59 |
|  | **15** | 31 | 4 | 1-4 | 3.06 | 1.53 |
|  | **Total** | **213** | **4** | **2-5** | **3.19** | **1.54** |
| **C. Primary and secondary prevention activities** | **1** | 36 | 4 | 3-5 | 3.94 | 1.31 |
|  | **2** | 39 | 4 | 2-5 | 3.47 | 1.48 |
|  | **3** | 35 | 4 | 3-5 | 3.60 | 1.35 |
|  | **13** | 36 | 3,5 | 2-5 | 3.30 | 1.60 |
|  | **14** | 36 | 4 | 3-5 | 3.75 | 1.38 |
|  | **15** | 31 | 4 | 3-5 | 3.68 | 1.35 |
|  | **Total** | **213** | **4** | **3-5** | **3.62** | **1.42** |

Note: the number of replies to each question, median, Q1-Q3 and the mean and SD are reported. The total values and values divided by district are reported in the table. In all questions, the maximum and minimum values correspond to the upper and lower limit of the Likert scale that is 5 and 1.

**Geospatial reference**

A map displaying the spatial distribution of the interviewed GPs/FPs’ main medical offices is shown in Figure S2.1.

**Figure S2.1.** Geospatial reference of the interviewed GPs/FPs’ main medical offices.
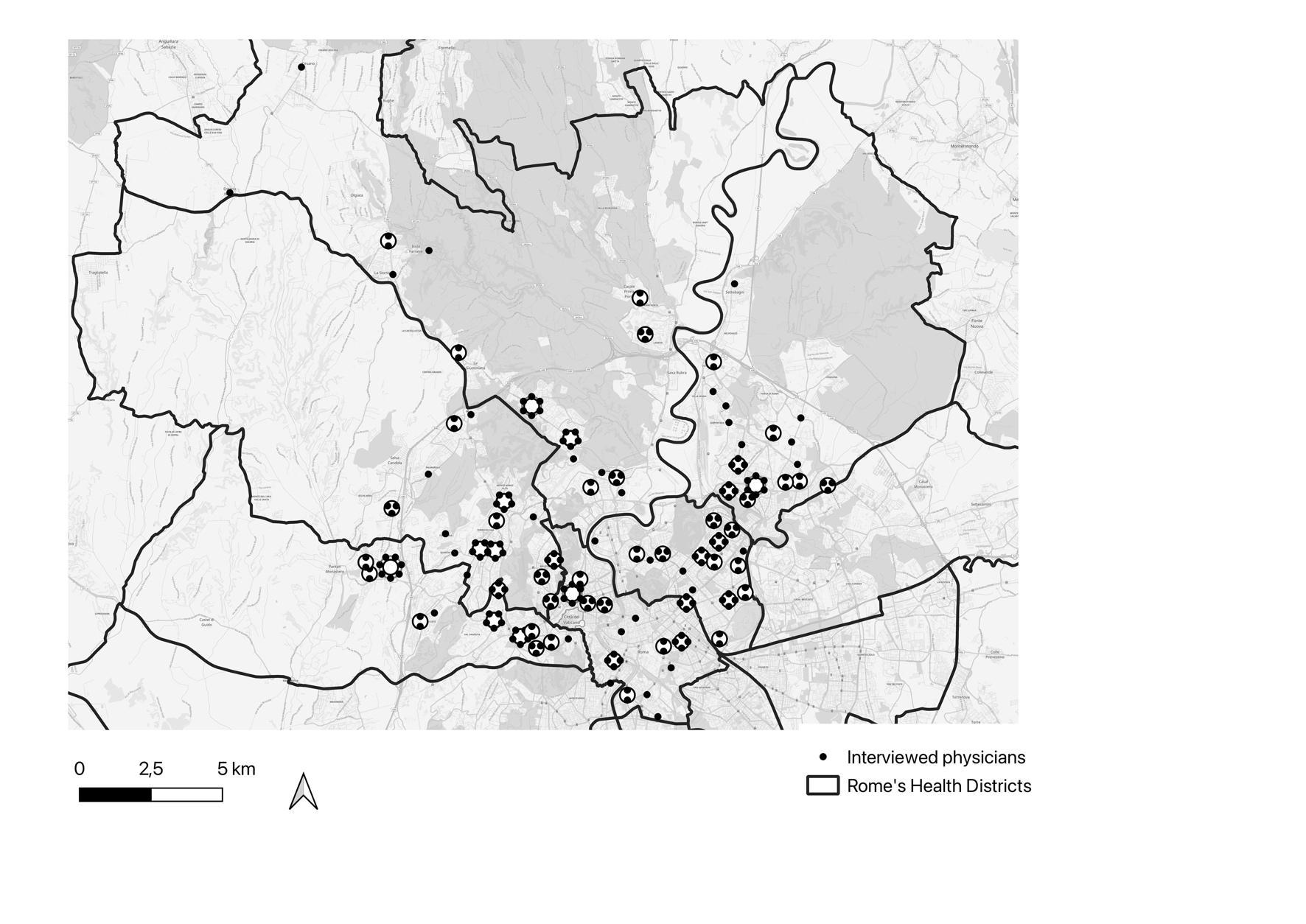


**References**

1. Gordon TJ. The Delphi method. Futures research methodology. 1994;2(3):1–33.

2. Hsu CC, Sandford BA. The Delphi Technique: Making Sense of Consensus. Practical Assessment, Research, and Evaluation. 2007;12:10.

3. Grime MM, Wright G. Delphi Method. Wiley StatsRef: Statistics Reference Online [Internet]. 2016 Aug 5 [cited 2022 Aug 12];1–6. Available from: https://onlinelibrary.wiley.com/doi/full/10.1002/9781118445112.stat07879

4. di Pietro ML, Zaçe D, Sisti LG, Frisicale EM, Corsaro A, Gentili A, et al. Development and validation of a questionnaire to assess Unaccompanied Migrant Minors’ needs (AEGIS-Q). Eur J Public Health [Internet]. 2021 Apr 24 [cited 2022 Aug 12];31(2):313–20. Available from: https://academic.oup.com/eurpub/article/31/2/313/5974958

5. Franco LM, Bennett S, Kanfer R. Health sector reform and public sector health worker motivation: a conceptual framework. Soc Sci Med [Internet]. 2002 [cited 2022 Aug 12];54(8):1255–66. Available from: https://pubmed.ncbi.nlm.nih.gov/11989961/

6. Krosnick JA, Presser S. Question and Questionnaire Design, in “Handbook of Survey Research.” In: Marsden P V., Wright JD, editors. 2nd ed. San Diego, CA: Elsevier; 2009.
